# Effects of forest disturbance on the fitness of an endemic rodent in a biodiversity hotspot

**DOI:** 10.1002/ece3.7214

**Published:** 2021-02-03

**Authors:** Olaoluwa John Ademola, Bram Vanden Broecke, Herwig Leirs, Loth S. Mulungu, Apia W. Massawe, Rhodes H. Makundi

**Affiliations:** ^1^ African Center of Excellence for Innovative Rodent Pest Management and Biosensor Technology Development Sokoine University of Agriculture Morogoro Tanzania; ^2^ Department of Wildlife Management Sokoine University of Agriculture Morogoro Tanzania; ^3^ Department of Zoology University of Ilorin Ilorin Nigeria; ^4^ Evolutionary Ecology Group Universiteit Antwerpen Wilrijk Belgium; ^5^ Pest Management Centre Sokoine University of Agriculture Morogoro Tanzania

**Keywords:** biodiversity hotspot, capture–mark–recapture, Eastern Arc Mountains, population density, *Praomys delectorum*, survival and maturation rate

## Abstract

*Praomys delectorum* occurs abundantly in both disturbed and intact forests in the Ukaguru Mountains within the Eastern Arc Mountains (EAM), Morogoro, Tanzania. While previous studies have reported that anthropogenic disturbances such as grazing, wood cutting, and harvesting have a positive effect on the population density of *P. delectorum*, the impact of habitat disturbance on its demographic traits is still unknown. We performed a capture–mark–recapture study in both disturbed and intact forests from June 2018 to February 2020 in order to investigate the effects of habitat disturbance on abundance and two demographic traits: survival and maturation of *P. delectorum* in the Ukaguru Mountains. We found no variation in abundance or maturation between intact and disturbed forests, but habitat type did affect survival. However, this effect was sex‐dependent since female survival was higher in disturbed forests, while male survival remained similar across the two forest types potentially due to differences in predation pressure or food availability between the two habitats. Continuous demographic monitoring of *P. delectorum* in EAM is necessary given that the increasing human population surrounding the landscape is leading to higher deforestation rates and expansion of the pine plantation in the forest reserve.

## Graphical abstract

In investigating the viability of the populations of a species, demographic parameters alongside population density should be considered. We found no variation in abundance or maturation of *Praomys delectorum* between intact and disturbed forests, but habitat type did affect survival. However, this effect was sex‐dependent since female survival was higher in disturbed forests, while male survival remained similar across the two forest types potentially due to differences in predation pressure or food availability between the two habitats.

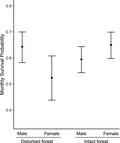

## 1 INTRODUCTION

Rodents, being the largest mammalian order, are well‐represented in sub‐Saharan Africa with 463 species adapted to heterogeneous environments and extent in all habitats and provide important ecosystem services (Monadjem et al., 2015). Rodents, and other small mammals in general, provide food for predators (ophidian, avian, and mammalian), regulate insect populations, and modify the soil (structure, organic content, and mineral cycling), which affects plant growth (Hayward & Phillipson, 1979). They consume and disperse seed (Hayward & Phillipson, 1979); for instance, in forest ecosystems, rodents are effective in seed dispersal by hoarding of seeds in caches which is a coping strategy for fluctuating seed supply (Corlett & Hughes, 2015). Nonetheless, most research on rodents in Africa has been focused on pest species, which are about 5%–17% of the African rodent species (Monadjem et al., 2015; Mulungu, 2017; Swanepoel et al., 2017) and data on nonpest species are rare. This research bias has potential consequences on the conservation of other, nonpest, rodent species in Africa (Swanepoel et al., 2017).

The Eastern Arc Mountains (EAM) region, being one of the top 25 biodiversity “hotspots” worldwide with at least 800 endemic vascular plants, and 136 endemic and 75 near‐endemic vertebrates, is facing an alarming rate of anthropogenic disturbance (Burgess et al., 1998, 2007; Myers et al., 2000; Rovero et al., 2014). One of these endemic vertebrates is the delectable soft‐furred mouse, *Praomys delectorum*, which occurs in moist montane forests of the EAM, and the distributional range extends westward to north‐central Tanzania, and southward to Malawi and northern Mozambique (Bryja et al., 2014; Cassola, 2016; Happold, 2013; Monadjem et al., 2015). However, this species is currently threatened by habitat loss due to deforestation and clearance of lands for agriculture throughout its distributional range (Cassola, 2016).

*Praomys delectorum* is a nocturnal, scansorial terrestrial rodent feeding on seeds, fruits, and insects found in burrows associated with the roots of large forest trees and under fallen wood (Happold, 2013; Monadjem et al., 2015). They are reported to be reproductively active during the late dry season and beginning of the wet season after which the population size increases with a peak at the end of the wet season and individuals surviving at most for 6 months (Happold, 2013). Information on the social and reproductive behavior of *P. delectorum* is scarce, though other species of the same genus appears to be territorial (Monadjem et al., 2015). *Praomys delectorum* is the dominant species in the Western Usambara Mountains in northeast Tanzania (Makundi et al., 2006), and habitat disturbance has been found to affect their feeding habits, reproduction, and parasitic infection rate in the Taita Hills, Kenya (Gitonga et al., 2015, 2016a, 2016b). Additionally, habitat disturbance has an effect on the population densities as well. Indeed, densities of *P. delectorum* have been reported to be higher in anthropogenically disturbed forest characterized by grazing, tree cutting, and wood collection (Cassola, 2016; Gitonga et al., 2015; Monadjem et al., 2015). This may suggest that this species is able to use resources in anthropogenically disturbed habitats. However, none of these studies looked at the demographic characteristics, which is key in order to understand the viability of the populations in disturbed habitats.

Indeed, while studying the population sizes of *P. delectorum* in disturbed and undisturbed habitat will undoubtedly provide valuable information for the conservation of this species, it is not sufficient. This is due to the fact that density alone is not a good estimator of the viability of the population since it does not take the individuals’ fitness into account (Van Horne, 1983). It is therefore important, in order to investigate the viability of the populations in disturbed habitats, to look at the demographic parameters that underlie these population dynamics (Oli & Dobson, 1999). Survival and maturation are two important components affecting the fitness of animals and are therefore indispensable in order to get a better understanding of their population dynamics. Indeed, estimating survival and maturation and combining these with the population density will provide us with more information about the impact of anthropogenic forest disturbance on *P. delectorum* populations.

Within this study, we investigated the effects of anthropogenic forest disturbance on *P. delectorum* population density as well as survival and maturation in the Ukaguru Mountains within the Eastern Arc Mountains, Tanzania. We hypothesize that population densities will be greater in anthropogenically disturbed forests characterized by grazing, tree cutting, and wood collection compared with undisturbed forests; because their feeding behavior has been reported to change in response to anthropogenic disturbances which may have positive effects on reproductive efforts and ultimately on population density size (Gitonga et al., 2015). Additionally, these changes in feeding behavior may also lead to a higher survival probability and maturation rate in disturbed areas compared with undisturbed forests as well. However, survival and maturation may vary between the wet and dry seasons, sexes, and age classes, as has been found in other small mammals (Eccard et al., 2002; Oli & Dobson, 1999; Previtali et al., 2010). Most research on the effects of habitat disturbance on the population dynamics in African small mammals focused on the pestiferous *Mastomys natalensis* (Julliard et al., 1999; Mayamba et al., 2019; Sluydts et al., 2007), and little information is available on *P. delectorum*. Our study will be the first to look at the effect of anthropogenic disturbance on both the population size and two demographic parameters of *P. delectorum* and is therefore important to fill this knowledge gap and will be useful to optimize the current conservation and management strategies of *P. delectorum* (Eberhardt, 1985; Oli & Dobson, 1999; Paradis et al., 1993).

## 2 MATERIAL AND METHODS

### 2.1 Study area

**FIGURE 1:**
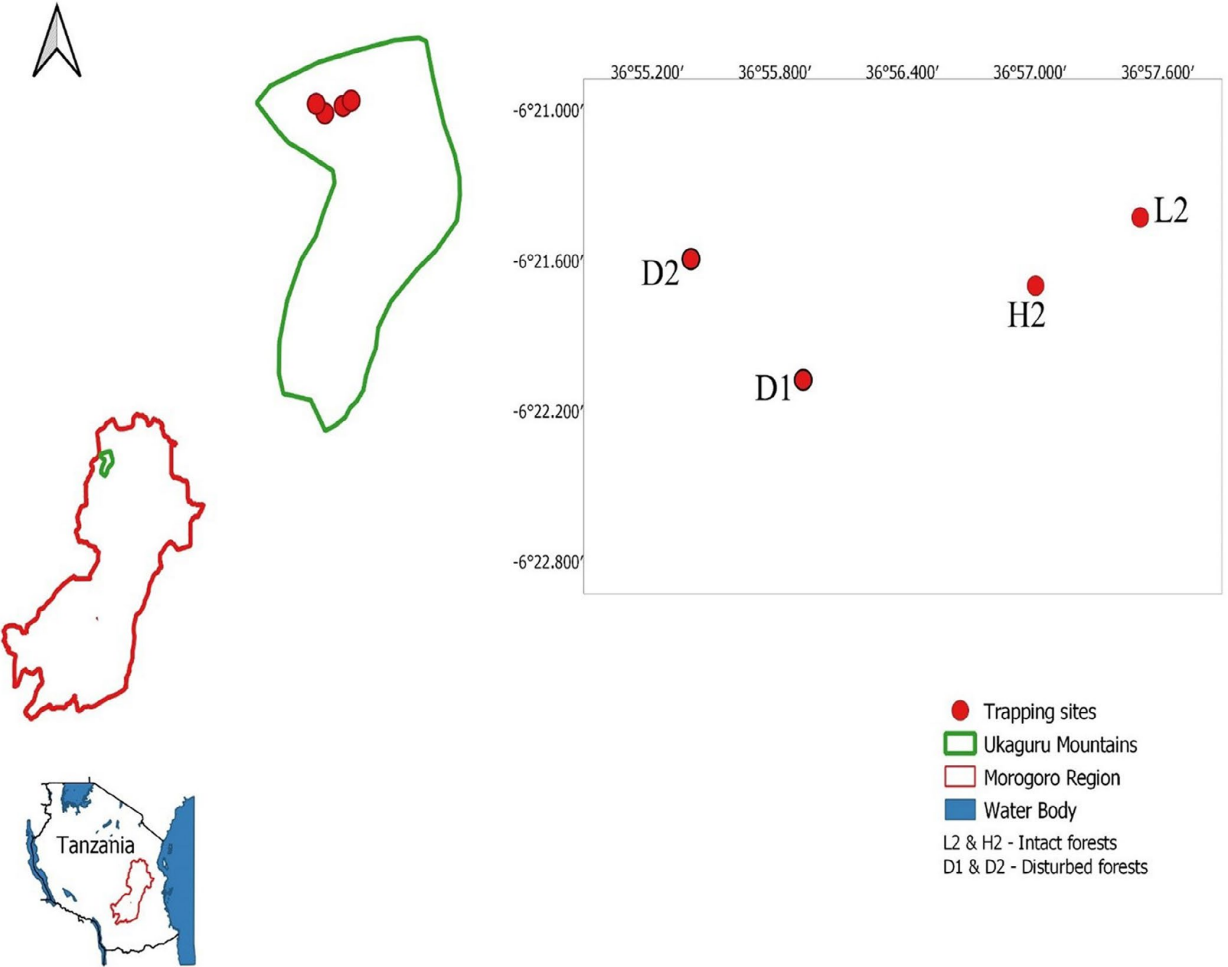
Study area and the coordinates of the trapping sites (map is not to scale)

This study was carried out in the Ukaguru Mountains within the Eastern Arc Mountains, located in the Gairo District, Morogoro, Tanzania (36°57′00″–38°00′00″ East and 06°25′00″–06°57′00″ South; Figure 1). The elevation of this landscape extends up to 2,250 m above sea level. The estimated annual rainfall is 1,400 mm (Gwegime et al., 2014). The dry season is between June and September, with maximum temperature of 21°C recorded in January and minimum temperature of 17°C in July at lower altitudes (Gwegime et al., 2014).

The vegetation type in the Ukaguru Mountains is montane and submontane forest. The montane forest is mainly characterized by the following tree species: *Polyscias fulva*, *Schefflera lukwangulensis*, *Garcinia volkensii*, *Ocotea usambarensis*, and *Cussonia spicata*. Others are *Dombeya burgessiae*, *Clerodendrum* sp., *Macaranga capensis*, and *Albizia gummifera*. The submontane forest is characterized by *Myrianthus holstii*, *Albizia gummifera*, *Allanblackia stuhlmannii*, and *Bersama abyssinica*. High forest disturbances observed include tree cutting, clearance of forest for agriculture, and grazing (Gwegime et al., 2014). The human population surrounding the forest is at least 75,720 people (Gwegime et al., 2014), and land outside the forest reserves is generally farmland. Crops commonly cultivated include pumpkin (*Cucurbita maxima*), banana (*Musa* spp), maize (*Zea mays*), Irish potatoes (*Solanum tuberosum*), common pea (*Pisum sativum*), beans (*Phaseolus lunatus*), and cedar (*Cedrela odorata*).

### 2.2 Trapping

Capture–mark–recapture (CMR) technique was used to trap rodents from June 2018 to February 2020. Two 70 × 70 m grids were set in intact sites (H2 and L2, 600 m apart), and two grids were placed in disturbed sites (D1 and D2, 600 m apart; Figure 1). The minimal distance (600 m) between the grids was sufficient to prevent migration between grids for small rodents. The two grids in the disturbed forest were in proximity (50 m) to human settlements and farmlands and were characterized by cattle grazing, illegal hunting, tree cutting, and wood collection. The two grids in the intact forests were devoid of human activities and were greater than 3 km from disturbed forests. Each grid consisted of seven parallel lines, 10 m apart, and seven trapping stations per line, also 10 m apart (a total of 49 trapping stations per grid). One Sherman LFA live trap (H.B Sherman Traps Inc.) was placed at each trapping station. Trapping of rodents was conducted for three consecutive nights every month. Traps were baited with peanut butter mixed with maize bran and inspected in the morning. The trapping station, sex, weight, and age were recorded. The reproductive status of captured animals was recorded, and the individuals were divided into two age classes based on their reproductive status: juveniles (not reproductive active) and adults (reproductive active). In males, the breeding condition was determined by position of the testes, whether scrotal or abdominal. In females, the breeding condition was determined either by signs of pregnancy by palpation, lactation, or perforate vagina (Makundi et al., 2006). Toe clipping (which does not affect survival of the animal) using number codes generated from CMR software *MARK* was employed in individual identification (Borremans et al., 2015). Captured animals were identified to species levels using relevant keys (Happold, 2013; Monadjem et al., 2015) and confirmed by sequencing the mitochondrial cytochrome b gene.

### 2.3 Statistical analysis

For analysis, we decided to focus only on *P. delectorum* since this was the most dominant species in all four fields (Table S1).

### 2.4 Population density

The population density of *P. delectorum* was calculated for each trapping session using the *M*(*h*) jackknife estimator in the DENSITY software (Version 5.0; Efford et al., 2004). However, this method is only useable when animals are captured for three consecutive nights, which was not always the case (even though we trapped for three nights). We therefore decided to use the minimal number of animals alive (MNA) as an alternative measurement for density. This method uses the individuals’ capture histories where we noted the individual as alive for all the trap sessions between the first and last time of capture.

In order to test for differences in abundance between the two forest types (disturbed and intact) and between seasons (dry and wet), we used a generalized linear mixed model with the minimal number of animals alive, calculated for each trapping session, as the response variable and a negative binomial error distribution (since there was evidence for over‐dispersion). We included season and forest type as fixed effects and allowed them to interact with each other. The field where the measurements were taken was included as a random effect. We excluded field L2 from the analysis since very few animals were captured during the whole study period (Figure 1; Table S1). The statistical analysis was executed using the R software 3.5.0 (R Core Team, 2013) with the glmmTMB package (version 1.0.2.1; Brooks et al., 2017). Differences in MNA between forest types and seasons were estimated using the effects package (version 4.1; Fox & Weisberg, 2019).

### 2.5 Goodness of fit

A goodness‐of‐fit (GOF) test was carried out with the U‐CARE software (Choquet et al., 2009; Choquet et al., 2009; Pradel et al., 2003) prior to the survival analysis to evaluate potential confounding factors such as an excess of transient animals and trap dependence. The test did not show any deviation against the assumption on transience (see results), which are individuals that were captured only once during the whole trapping period. Additionally, the GOF test revealed no effect of trap dependence (see results), which suggests that the recapture probability of the individuals did not depend on previous trapping experience.

### 2.6 Survival and maturation analysis

Survival and recapture probabilities were estimated using a multivariate multistate Cormack–Jolly–Seber model in E‐SURGE V2.1.4 (Choquet, Lebreton, et al., 2009; Choquet, Rouan, et al., 2009). This allowed us to estimate the effect of age (adult or juvenile), sex (male or female), and forest type (disturbed or intact forest) on both survival (*φ*) and maturation (Ψ) probabilities. We included three events (captured as adult/juvenile or not captured at all) and three states (captured as an adult or juvenile or not captured at all). Trapping was done using Pollock's closed robust design, where the population is assumed to be closed (i.e., no entry or exit of individuals into the population) within each trap session and open between trap sessions. Survival was therefore defined as the probability to survive from 1 month to the next and fixed to 1 within a trapping session, while the recapture probability was estimated within each session.

Survival and maturation probabilities were modeled in subsequent steps which reduced the amount of models that we needed to run. We first modeled survival after which we modeled maturation (Mariën et al., 2018; Mayamba et al., 2019; Sluydts et al., 2007). Models were ranked using the sample size corrected Akaike's information criterion (AIC; Burnham & Anderson, 2004), where the model with the lowest AIC value was the best fit for the data and selected as starting point for the next modeling step. Models that differed less than 2.0 units were deemed equally good.

#### 2.6.1 Survival

Before we started with actual model reduction, we needed to test whether there was seasonal variation in survival (Table 1: seasonal effects). We therefore created three models where we allowed survival to vary either (a) between the two seasons separately for each year (season × year: dry season: June 2018–September 2018, wet season: October 2018–May 2019, dry season: June 2019–September 2019, wet season: October 2019–February 2020), (b) between the wet and dry seasons but compiling the 2 years together (season: dry vs. wet season), or (c) by creating a model without seasonality (Table 1). Within these three models, we allowed survival to vary between the two age classes (adults and juvenile) and between males and females separately for intact and disturbed forests, since we allowed sex and age to interact with forest type (Table 1). We then selected, out of these three model, the model with the lowest AIC as a starting point for further model reduction. This was done in two substeps, where we first removed all the interactions between forest type and age and sex one by one until the three covariates (sex, age, and forest type) had an additive effect (Table 1: reduction interactions). We then chose, out of these models, the model with the lowest AIC value as a starting point for the second substep, where we stepwise remove each covariate one by one until all three of them were removed (Table 1: reduction fixed effects). The model with the lowest AIC value, after this final step, was considered to be best fitted model concerning the survival within this study.

**TABLE 1:** Modeling of survival and maturation. Highlighted models (bold) were selected in each step and used a starting point in subsequent steps

Model	Survival	Maturation	Np	Deviance	AIC	ΔAIC
(1) Survival						
Seasonal effects						
	**F** × **(A + S)**	**i**	**32**	**5,706.51**	**5,770.51**	**0.00**
	Season × [F × (A + S)]	i	38	5,701.09	5,777.09	6.58
	Season × year × [F × (A + S)]	i	50	5,691.01	5,791.01	20.50
Reduction interactions						
	**F** × **S + A**	**i**	**31**	**5,706.51**	**5,768.51**	**0.00**
	F × (A + S)	i	32	5,706.51	5,770.51	2.00
	F + S + A	i	30	5,714.02	5,774.02	5.50
	F × A + S	i	31	5,713.69	5,775.69	7.18
Reduction: fixed effects						
	**F** × **S**	**i**	**30**	**5,706.81**	**5,766.81**	**0.00**
	F × S + A	i	31	5,706.51	5,768.51	1.70
	i	i	27	5,714.76	5,768.76	1.95
	A	i	28	5,714.36	5,770.36	3.55
	F	i	28	5,714.41	5,770.41	3.59
	S	i	28	5,714.76	5,770.76	3.95
	F + A	i	29	5,714.05	5,772.05	5.24
	S + A	i	29	5,714.35	5,772.35	5.53
	F + S	i	29	5,714.40	5,772.40	5.59
(2) Maturation						
	**F** × **S**	**S**	**31**	**5,704.20**	**5,766.20**	**0.00**
	F × S	i	30	5,706.81	5,766.81	0.61
	F × S	F	31	5,705.27	5,767.27	1.07
	F × S	F + S	32	5,703.40	5,767.40	1.20
	F × S	F × S	33	5,701.44	5,767.44	1.25

Note

For each model, the number of parameters (Np), deviance, and AIC are given. ΔAIC is the difference in AIC between the respective model and the top‐ranked one. Each model was run with the same recapture probabilities. Abbreviations: S, sex (male or female); A, age (adult or juvenile); F, forest type (disturbed and intact forest); i, intercept; season (wet and dry season); season × year (dry season: June 2018–September 2018, wet season: October 2018–May 2019, dry season: June 2019–September 2019, wet season: October 2019–February 2020).

Models are ranked on the AIC from low to high.

#### 2.6.2 Maturation

After survival, we modeled maturation which is defined as the monthly probability for juveniles to become adults, that is, to become reproductive active since adults and juveniles were differentiated from each other based on signs of sexual activity. We started the model reduction from a full model where maturation rate was allowed to differ between the two sexes within each forest type (Table 1). We then removed the interaction and all covariates one by one; only the intercept model remained (Table 1). The model with the lowest AIC value was the best fit for the data.

Since variation in survival and maturation probabilities is the main focus of this work, we decided to use the same recapture parameters in every model. Recapture probability was fully time‐dependent and was allowed to differ between the four different fields.

### 2.7 Ethical considerations

This research was approved by the Sokoine University of Agriculture, Tanzania (reference: SUA/DPRTC/PFC/D/2017/0010/11) and Tanzania Forest Service Agency (TFS). Animal handling followed the guidelines of the American Society of Mammalogists (ASM) for the use of wild mammals in research and education (Sikes & Animal Care and Use Committee of the American Society of Mammalogists, 2016).

## 3 RESULTS

### 3.1 Population dynamics

**FIGURE 2:**
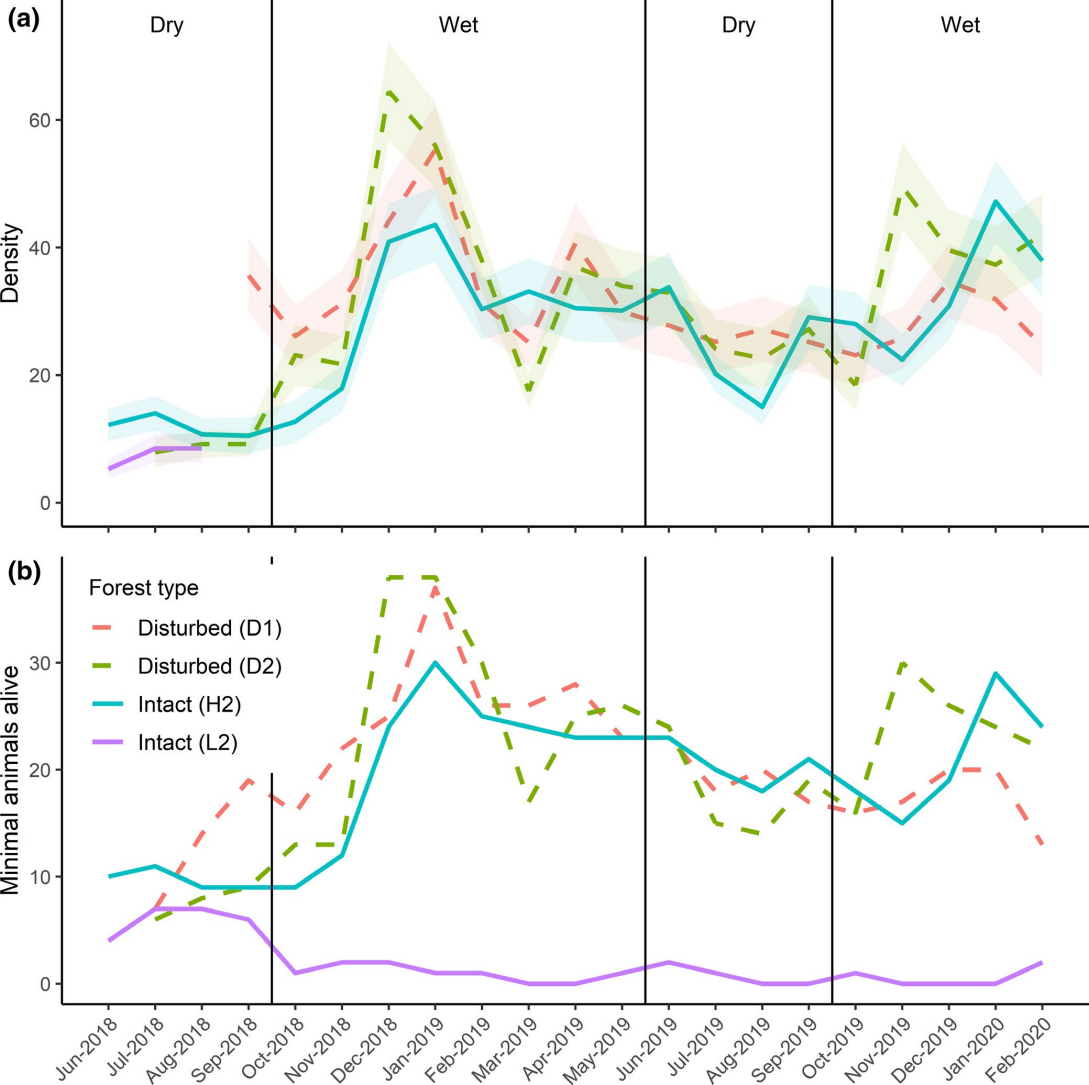
Population size of *Praomys delectorum* in both disturbed forests (dashed lines: field D1: red and D2: green) and intact forests (solid lines: field H2: blue and L2: purple) calculated using the two methods: (a) the *M*(*h*) jackknife estimator (b) the minimal number of animals alive

**FIGURE 3:**
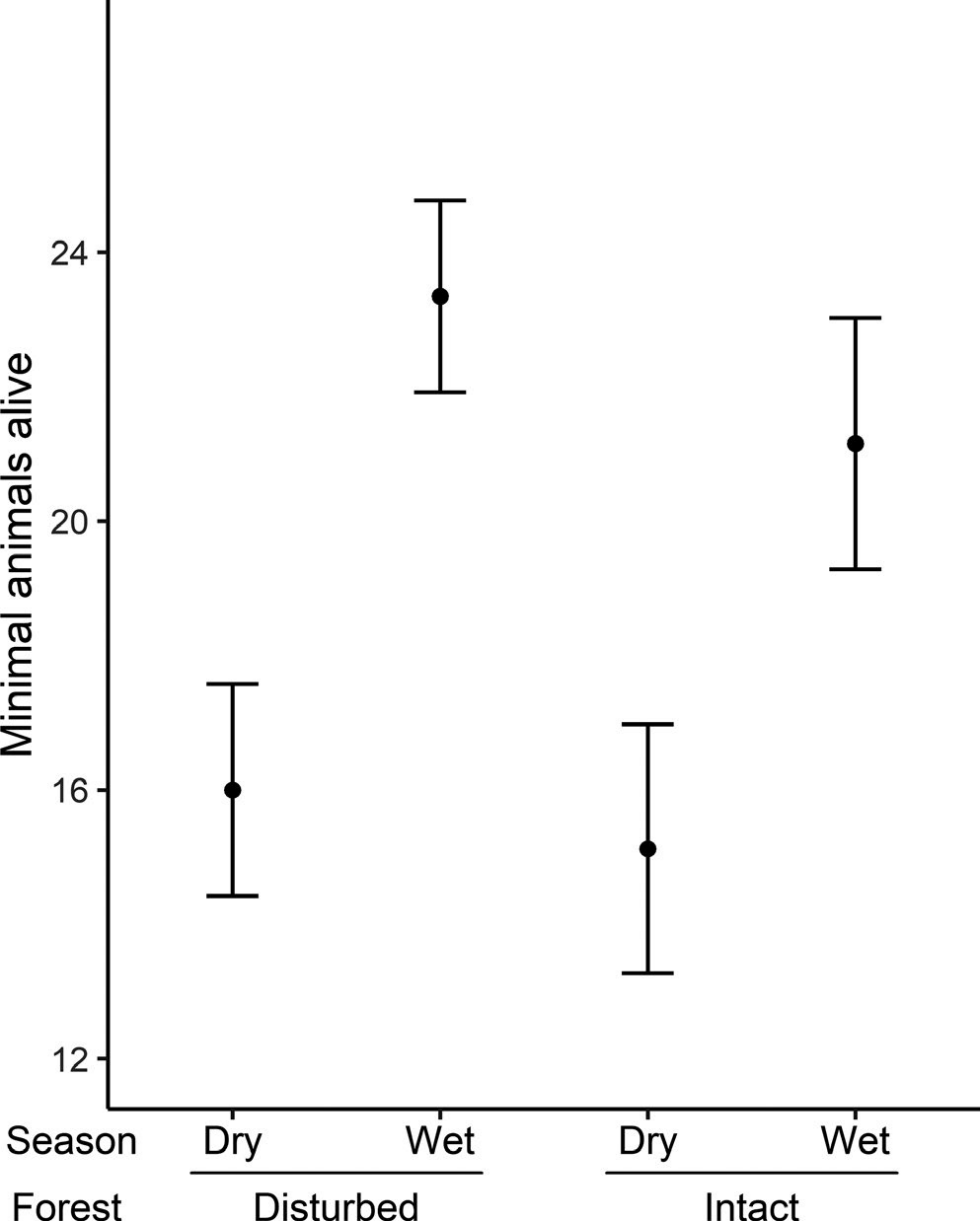
Predicted mean of the minimal number of animals alive for *Praomys delectorum* derived from the generalized linear mixed model during the dry and wet seasons within both the disturbed and intact forests with their standard errors

Population densities of *P. delectorum* as derived using the *M*(*h*) estimator and MNA showed concordance. The population density of *P. delectorum* varied temporally with peaks attained in the wet seasons in both disturbed and intact forests (Figure 2). The generalized linear mixed model revealed that *P. delectorum* abundance was significantly higher during the wet season compared with the dry season (estimate ± *SE*: 0.378 ± 0.116, Z = 3.257, *p* = 0.001; Figure 3). There was no significant differences between the two forest types (−0.056 ± 0.157, *Z* = −0.357, *p* = 0.721), and the interaction was also not significant (−0.042 ± 0.191, *Z* = −0.222, *p* = 0.824).

### 3.2 Goodness of fit

The GOF test revealed no deviation against the assumption of transience (Test 3G.SR: *χ* ^2^ = 106.590, *df* = 96, *p* = 0.216), not against trap dependence (Test M.ITEC, *χ* ^2^ = 80.173, *df* = 67, *p* = 0.130). This suggesting that there was no excess of animals that were trapped only once during the study period and that there was no trap effect, in which the individuals became trap happy or shy when they were trapped during the previous night.

### 3.3 Model selection

#### 3.3.1 Survival

**FIGURE 4:**
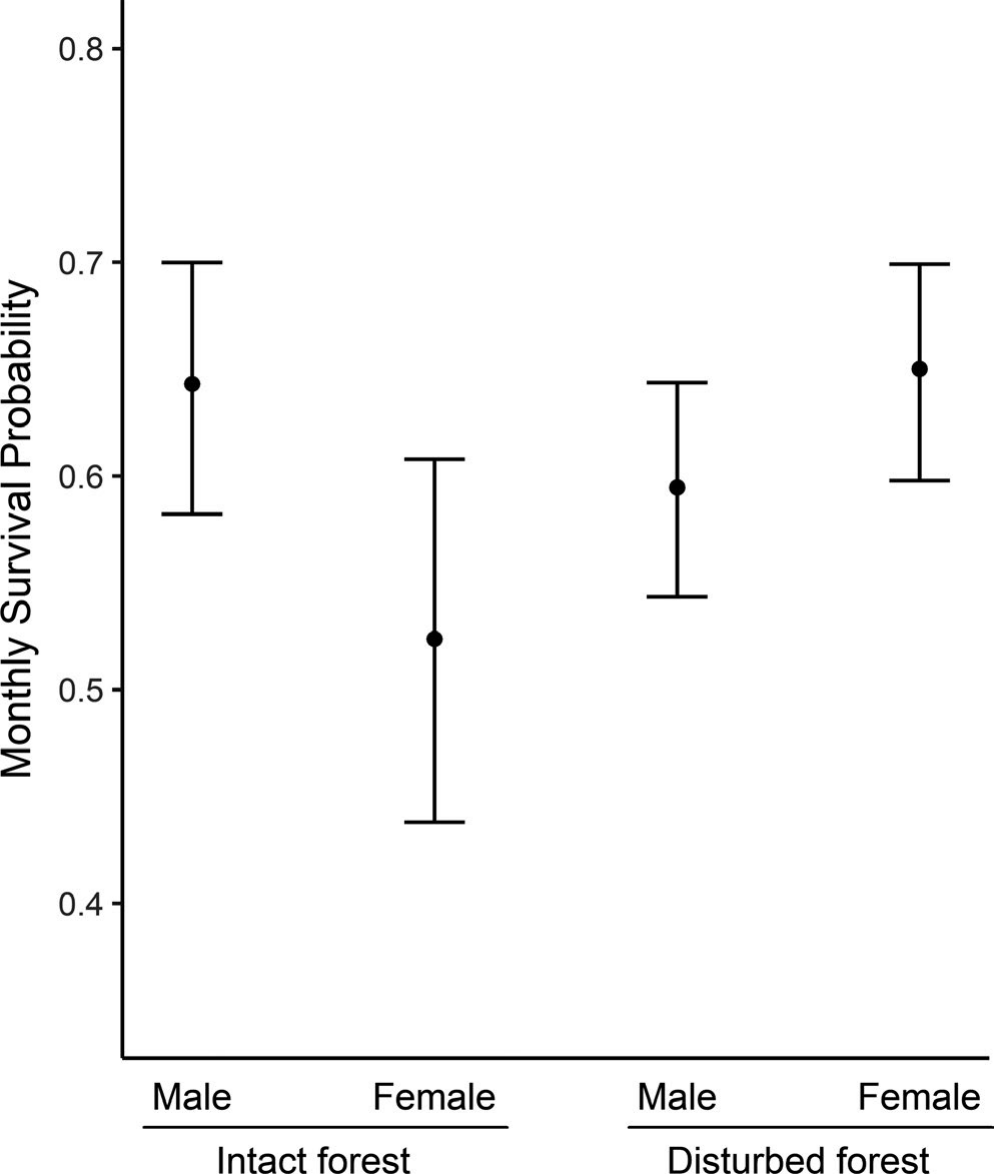
Monthly survival probability of male and female *Praomys delectorum* in disturbed and intact forests derived from the best fitted model. Females had an increased survival probability in disturbed forests compared with intact forests, while male had a similar survival probability in both forest types. Error bars represent the 95% confidence interval

We first studied whether there were differences in survival between the different seasons by comparing two models with a seasonal effect (with and without time dependence) and one without a seasonal component. The model without a seasonal component had a significant lower AIC value compared with the other two models with a season component, which suggests that *P. delectorum* survival does not change between seasons (Table 1). The highest ranking model (with the lowest AIC value; Table 1) revealed differences in survival between the disturbed and intact forests, but this was sex‐specific. Indeed, the model showed that female survival was higher in disturbed forests (estimate ± *SE*: 0.650 ± 0.026) compared with intact forests (0.524 ± 0.044), while male survival was slightly lower in disturbed forest (0.595 ± 0.026) compared with intact forest (0.643 ± 0.030; Figure 4). The second‐best model was 1.700 units larger compared with the first model and had age as an additional additive effect (Table 1), where juvenile survival was always higher compared with adults for both females (disturbed forest: juveniles = 0.658 ± 0.030, adults = 0.640 ± 0.032; intact forest: juveniles 0.534 ± 0.047, adults = 0.514 ± 0.047) as males (disturbed forest: juveniles = 0.608 ± 0.035, adults = 0.589 ± 0.028; intact forest: juveniles 0.656 ± 0.038, adults = 0.638 ± 0.032) in both forest types. However, these differences in survival between adults and juveniles were small, and we decided to continue with the model with the lowest AIC value.

#### 3.3.2 Maturation

The model with the lowest AIC value contained sex (Table 1) where males matured faster (0.090 ± 0.015) compared with females (0.060 ± 0.011). However, the second‐best model had only an intercept (0.073 ± 0.009) and was only 0.61 AIC units larger compared with the first model (Table 1), which suggests that these differences in maturation rate between males and females are not strongly supported.

#### 3.3.3 Recapture probability

Each model ran with the same recapture parameters. The models showed that the recapture probability varied over time and that the recapture probability differed between the four fields (Figure S1).

## 4 DISCUSSION

Habitat disturbances due to anthropogenic activities have been found to affect survival and maturation of several vertebrate species (e.g., birds, rodents; Borges & Marini, 2010; Cosset et al., 2018; Korfanta et al., 2012), but this is the first study that looked at this effect on *P. delectorum*. We found that *P. delectorum* was the most dominant species in the Ukaguru Mountains in both disturbed and intact forests and that their density varied seasonally, being significantly greater during the wet season compared with the dry season. While forest disturbance had no effect on abundance or maturation, it did affect female survival, which was higher in disturbed forests compared with intact forests.

*Praomys delectorum* was the most dominant species in both disturbed and intact forests of the Ukaguru Mountains which is in line with other studies in montane forests in East Africa (Gitonga, 2007; Gitonga et al., 2015, 2016a; Makundi et al., 2006). However, the abundance of *P. delectorum* varied seasonally, with significantly higher densities during the wet season compared with the dry season, which is similar to the findings of Makundi et al. (2006). This may suggest that *P. delectorum* exhibits a seasonal breeding pattern which starts at the beginning of the wet season (Happold, 2013). Indeed, rainfall has been shown to have a large effect on the timing of the breeding season in a wide variety of small mammals, since it affects the availability of food (Field, 1975; Leirs et al., 1989; Taylor & Green, 1976) allowing the population to grow until food becomes more scarcely available at the beginning of the dry season leading to a decrease in the population size (Leirs et al., 1994). The reason for the low captures in field L2 is not clear. We suspect that the noise generated from tree felling in an adjacent pine plantation using chain saws and skidding may have shifted the home range of populations in this field. This experience was only peculiar to L2. Indeed, exposure of rodents to noise leads to stress induction (Baldwin, 2007), and they may respond by fleeing farther away (Hawthorne et al., 2011).

However, we found no differences in abundance between the disturbed and intact forests, which contradicts the general idea that members of the genus *Praomys* are more abundant in disturbed habitats than unperturbed habitats (Cassola, 2016; Monadjem et al., 2015). Indeed, *P. delectorum* was reported to occur at higher densities in disturbed habitats compared with intact forests in Taita hills, Kenya (Gitonga et al., 2015, 2016a), potentially because these disturbed habitats are characterized by a lower predation risks (Lambert et al., 2003) and a higher availability of food resources (Gitonga et al., 2015, 2016a; Ochoa, 2000). It is currently unclear why our results deviate from this general assumption. A potential explanation is that using the minimal number of animals alive (MNA) as a proxy for density might have caused a bias in our data, since it does not take individual variation in capture probability into account (Pocock et al., 2004). However, this explanation seems unlikely since the results from the MNA were similar to that from the *M*(*h*) jackknife estimator which takes variability in capture probabilities among individuals into account (Burnham & Overton, 1978). Alternatively, this may be the case of populations of the same species from different geographical regions responding differently to habitat disturbances (Frederiksen et al., 2005).

Nevertheless, density alone is not sufficient to conclude on the viability of the populations in both disturbed and intact forests. Our models revealed a higher survival probability in disturbed forest, but only for females, while male survival remained similar between both forest types. This might result from differences in either predation pressure or resource availability between the two forest types which may act stronger on females than males. Small carnivores are probably the most important predators of *P. delectorum* and selectively prey on females, since they are less mobile than males when pregnant or because of the scent and noises of their young reveal the location of their burrows (Happold, 2013; Korpimaki, 1985; Norrdahl & Korpimaki, 1998). However, the predation pressure on small mammals decreases in disturbed habitats since these predators are less abundant and diverse in anthropogenic disturbed habitats (Lambert et al., 2003, 2006), potentially due to an increased mortality rate (Bonnet et al., 1999), which may explain the higher survival rate of females in these habitats. However, more studies are required to show the impact of predators on this species in this landscape.

An alternative, nonmutually exclusive, explanation is variation in food availability between disturbed and intact forests. While food availability is vital for the survival of all rodents (Kennis et al., 2012), females have been found to depend more heavily on food acquisition than males (Ostfeld, 1985). Survival of female Californian voles (*Microtus californicus*), for example, depended more heavily on the spatial and seasonal distribution on resources compared with males (Ostfeld, 1985). This may explain why female survival is higher in disturbed forests compared with intact forests, since food availability is considered to increase with habitat disturbance (Greenberg et al., 2011; Ochoa, 2000; Zhang et al., 2009). Indeed, forest disturbance has been found to alter plant communities (Hawthorne et al., 2011) and to stimulate hoarding efforts by seed‐caching rodents (Greenberg et al., 2011; Zhang et al., 2009).

While habitat disturbance affected survival, maturation remained similar between disturbed and intact forests. This may stem from the fact that food is continuously available in both habitat types. Both Leirs et al. (1997) and Sluydts et al. (2007) have shown that maturation rate in *M. natalensis* correlated positively with preceding cumulative rainfall which triggered greater food availability. The finding of the current study is consistent with Mayamba et al. (2019) who reported that habitat did not affect maturation of *M. natalensis* in Uganda; the animals had continuous access to food resources and in no way was their normal growth and development impeded. Another best supported model showed maturation rate of females to be lower compared with males which may be due to response to pre‐ or postnatal stress or secretion of puberty‐delaying pheromones in females (Oli & Dobson, 1999). However, longer trapping period is required to unravel which factors influence maturation rate in *P. delectorum*.

Population dynamics are driven by demographic parameters with some of these parameters acting on the population greatly than others (Oli & Dobson, 1999). In this study, whereas the population densities of *P. delectorum* varied seasonally, with significantly higher densities during the wet season compared with the dry season, survival and maturation rates were not seasonal in both forest types and therefore may not be the underlying demographic mechanisms responsible for such temporal changes in abundance. To account for the temporal variation in population density of *P. delectorum*, there is a need to investigate the effects of predation and other demographic parameters such as reproduction, recruitment, and movement (dispersal). Populations of *P. delectorum* may be stable and viable in this landscape in spite of forest disturbances as indicated in the insignificant differences in population sizes between both forest types, and greater survival rate in disturbed forests. Also, this does not imply that forest disturbance should be left unchecked in this landscape as unperturbed forests are irreplaceable in the conservation of biodiversity (Gibson et al., 2011). Though our findings suggest that forest disturbance affects the survival rate of *P. delectorum*, we recommend further long‐term studies in order to arrive at strong conclusions. The IUCN Least Concern conservation status of *P. delectorum* (Cassola, 2016) is uncertain given the very frequent rates of anthropogenic disturbances in the EAM. Therefore, continuous demographic monitoring of *P. delectorum* in EAM is necessary given that human populations surrounding the landscape are increasing leading to deforestation and expansion of the pine plantation in the forest reserve.

## CONFLICT OF INTEREST

The authors declare that they have no conflict of interest.

## AUTHOR CONTRIBUTIONS

**Olaoluwa John Ademola:** Conceptualization (lead); data curation (lead); formal analysis (equal); investigation (lead); methodology (lead); project administration (lead); resources (lead); writing – original draft (lead); writing – review and editing (equal). **Bram Vanden Broecke:** Formal analysis (equal); writing – review and editing (equal). **Herwig Leirs:** Formal analysis (equal); writing – review and editing (equal). **Loth S. Mulungu:** Project administration (equal); resources (equal); supervision (equal); writing – review and editing (equal). **Apia W. Massawe:** Funding acquisition (lead); project administration (equal); resources (equal); supervision (equal); writing – review and editing (equal). **Rhodes H. Makundi:** Conceptualization (equal); funding acquisition (lead); project administration (lead); resources (equal); supervision (equal); writing – review and editing (equal).

## Supporting information

Figure S1

Table S1

Supplementary Material

## Data Availability

We, the authors of this manuscript, have deposited the data used in the result section to public domain Dryad. https://doi.org/10.5061/dryad.j6q573ncr.

## References

[ece37214-bib-0001] Baldwin, A. L. (2007). Effects of noise on rodent physiology. International Journal of Comparative Psychology, 20, 134–144.

[ece37214-bib-0002] Bonnet, X. , Naulleau, G. , & Shine, R. (1999). The dangers of leaving home: Dispersal and mortality in snakes. Biological Conservation, 89, 39–50. 10.1016/S0006-3207(98)00140-2

[ece37214-bib-0003] Borges, F. J. A. , & Marini, M. A. (2010). Birds nesting survival in disturbed and protected Neotropical savannas. Biodiversity and Conservation, 19, 223–236. 10.1007/s10531-009-9718-z

[ece37214-bib-0004] Borremans, B. , Sluydts, V. , Makundi, R. H. , & Leirs, H. (2015). Evaluation of short‐, mid‐ and long‐term effects of toe clipping on a wild rodent. Wildlife Research, 42, 143–148. 10.1071/WR14109

[ece37214-bib-0005] Brooks, M. E. , Kristensen, K. , Benthem, K. J. , Magnusson, A. , Berg, C. W. , Nielsen, A. , Skaug, H. J. , Mächler, M. , & Bolker, B. M. (2017). glmmTMB balances speed and flexibility among packages for zero‐inflated generalized linear mixed modeling. R Journal, 9(2), 378–400. 10.32614/RJ-2017-066

[ece37214-bib-0006] Bryja, J. , Mikula, O. , Patzenhauerová, H. , Oguge, N. O. , Šumbera, R. , & Verheyen, E. (2014). The role of dispersal and vicariance in the Pleistocene history of an East African mountain rodent, *Praomys delectorum* . Journal of Biogeography, 41(1), 196–208. 10.1111/jbi.12195

[ece37214-bib-0007] Burgess, N. D. , Butynski, T. M. , Cordeiro, N. J. , Doggart, N. H. , Fjeldså, J. , Howell, K. M. , Kilahama, F. B. , Loader, S. P. , Lovett, J. C. , Mbilinyi, B. , Menegon, M. , Moyer, D. C. , Nashanda, E. , Perkin, A. , Rovero, F. , Stanley, W. T. , & Stuart, S. N. (2007). The biological importance of the Eastern Arc Mountains of Tanzania and Kenya. Biological Conservation, 134(2), 209–231. 10.1016/j.biocon.2006.08.015

[ece37214-bib-0008] Burgess, N. D. , Fjeldså, J. , & Botterweg, R. (1998). Faunal importance of the Eastern Arc Mountains of Kenya and Tanzania. Journal of East African Natural History, 87(1), 37–58. 10.2982/0012-8317(1998)87[37:FIOTEA]2.0.CO;2

[ece37214-bib-0009] Burnham, K. P. , & Anderson, D. R. (2004). Multimodel Inference: understanding AIC and BIC in model selection. Sociological Methods & Research, 33, 261–304. 10.1177/0049124104268644

[ece37214-bib-0010] Burnham, K. P. , & Overton, W. S. (1978). Estimation of the size of a closed population when capture probabilities vary among animals. Biometrika, 65(3), 625–633. 10.1093/biomet/65.3.625

[ece37214-bib-0011] Cassola, F. (2016). Praomys delectorum. The IUCN Red List of Threatened Species 2016: e.T18114A22418403. 10.2305/IUCN.UK.2016-2.RLTS.T18114A22418403.en. Accessed May 1, 2020.

[ece37214-bib-0012] Choquet, R. , Lebreton, J.‐D. , Gimenez, O. , Reboulet, A.‐M. , & Pradel, R. (2009). U‐CARE: Utilities for performing goodness of fit tests and manipulating CApture REcapture data. Ecography, 32(6), 1071–1074. 10.1111/j.1600-0587.2009.05968.x

[ece37214-bib-0013] Choquet, R. , Rouan, L. , & Pradel, R. (2009). Program E‐surge: A software application for fitting multievent models. In D. L. Thomson , E. G. Cooch , & M. J. Conroy (Eds.), Modeling demographic processes in marked populations. *Environmental and Ecological Statistics*, Vol. 3. Springer. 10.1007/978-0-387-78151-8_39

[ece37214-bib-0014] Corlett, R. T. , & Hughes, A. C. (2015). Mammals in forest ecosystems. In K.‐S.‐H. Peh , R. T. Corlett , & Y. Bergeron (Eds.), The Routledge handbook of forest ecology (pp. 264–278). Routledge.

[ece37214-bib-0015] Cosset, C. C. P. , Gilroy, J. J. , & Edwards, D. P. (2018). Impacts of tropical forest disturbance on species vital rates. Conservation Biology, 33(1), 66–75. 10.1111/cobi.13182 29972268

[ece37214-bib-0016] Eberhardt, L. L. (1985). Assessing the dynamics of wild populations. The Journal of Wildlife Management, 49(4), 997–1012. 10.2307/3801386

[ece37214-bib-0017] Eccard, J. A. , Klemme, I. , Horne, T. J. , & Hannu, Y. (2002). Effects of competition and season on survival and maturation of young bank vole females. Evolutionary Ecology, 16, 85–99. 10.1023/A

[ece37214-bib-0018] Efford, M. , Dawson, D. , & Robbins, C. (2004). DENSITY: Software for analysing capture‐recapture data from passive detector arrays. Animal Biodiversity and Conservation, 27(1), 217–228.

[ece37214-bib-0019] Field, A. C. (1975). Seasonal changes in reproduction, diet and body composition of two equatorial rodents. East African Wildlife Journal, 13, 221–235. 10.1111/j.1365-2028.1975.tb00136.x

[ece37214-bib-0020] Fox, J. , & Weisberg, S. (2019). An R companion to applied regression (3rd ed.). Sage.

[ece37214-bib-0021] Frederiksen, M. , Harris, M. P. , & Wanless, S. (2005). Inter‐population variation in demographic parameters: A neglected subject? Oikos, 2, 209–214. 10.1111/j.0030-1299.2005.13746.x

[ece37214-bib-0022] Gibson, L. , Lee, T. M. , Koh, L. P. , Brook, B. W. , Gardner, T. A. , Barlow, J. , Peres, C. A. , Bradshaw, C. J. A. , Laurance, W. F. , Lovejoy, T. E. , & Sodhi, N. S. (2011). Primary forests are irreplaceable for sustaining tropical biodiversity. Nature, 478(7369), 378–381. 10.1038/nature10425 21918513

[ece37214-bib-0023] Gitonga, J. W. (2007). Effect of habitat fragmentation on food habits, intestinal parasites and aspects of reproduction among *Praomys delectorum* sub‐populations in the Taita and Kyulu hills. Kenyatta University.

[ece37214-bib-0024] Gitonga, J. , Simbauni, J. , & Oguge, N. (2015). Effect of habitat fragmentation on food habits and gastrointestinal tract of *Praomys delectorum* sub‐populations in the Taita and Kyulu hills. Kenya. International Journal of Current Research, 4(1), 63–72.

[ece37214-bib-0025] Gitonga, J. , Simbauni, J. , & Oguge, N. (2016a). Effect of habitat fragmentation on some aspects of reproduction among *Praomys delectorum* sub‐populations in the Taita and Kyulu hills, Kenya. International Journal of Advanced Research in Biological Sciences, 3(4), 143–151.

[ece37214-bib-0026] Gitonga, J. , Simbauni, J. , & Oguge, N. (2016b). Habitat fragmentation and occurrence of intestinal parasites among the *Praomys delectorum* sub‐populations in Taita and Kyulu hills, Kenya. South Indian Journal of Biological Sciences, 2(3), 354–359. 10.22205/sijbs/2016/v2/i3/100301

[ece37214-bib-0027] Greenberg, C. H. , Perry, R. W. , Harper, C. A. , Levey, D. J. , & Mccord, J. M. (2011). The role of young, recently disturbed upland hardwood forest as high quality food patches. In C. H. Greenberg , B. Collins , & F. Thompson (Eds.), Sustaining young forest communities: Ecology and management of early successional habitats in the Central Hardwood Region USA (pp. 121–141). 10.1007/978-94-007-1620-9

[ece37214-bib-0028] Gwegime, B. J. , Mwangoka, M. , Mulungu, E. , Latham, J. , Gereau, R. E. , & Doggart, N. (2014). The biodiversity and forest condition of Mamiwa‐Kisara North Forest Reserve. TFCG Technical Paper 41. DSM, Tz. pp. 1–86.

[ece37214-bib-0029] Happold, D. C. D. (Ed.) (2013). Mammals of Africa. Volume III: Rodents, hares and rabbits (pp. 524–525). Bloomsbury Publishing.

[ece37214-bib-0030] Hawthorne, W. D. , Marshall, C. A. M. , Juam, M. A. , & Agyeman, V. K. (2011). The impact of logging damage on tropical rainforests, their recovery and regeneration an annotated bibliography. Oxford.

[ece37214-bib-0031] Hayward, G. F. , & Phillipson, J. (1979). Community structure and functional role of small mammals in ecosystems. In D. M. Stoddart (Ed.), Ecology of small mammals (pp. 135–212). Chapman and Hall.

[ece37214-bib-0032] Julliard, R. , Leirs, H. , Chr, N. , Yoccoz, N. G. , Caroline, A. , Vot, P. R. E. , & Verheyen, W. (1999). Survival‐variation within and between functional categories of the African multimammate rat. Journal of Animal Ecology, 68, 550–561. 10.1046/j.1365-2656.1999.00304.x

[ece37214-bib-0033] Kennis, J. , Laurent, C. , Amundala, N. D. , Migimiru, A. , & Leirs, H. (2012). Survival and movement of the Congo forest mouse (*Deomys ferrugineus*): A comparison of primary rainforest and fallow land in Kisangani. Democratic Republic of Congo. African Zoology, 47(1), 147–159. 10.1080/15627020.2012.11407533

[ece37214-bib-0034] Korfanta, N. M. , Newmark, W. D. , & Kauffman, M. (2012). Understory bird community long‐term demographic consequences of habitat fragmentation to a tropical understory bird community. Ecology, 93(12), 2548–2559. 10.2307/41739613 23431586

[ece37214-bib-0035] Korpimaki, E. (1985). Prey choice strategies of the kestrel *Falco tinnunculus* in relation to available small mammals and other Finnish birds of prey. Annales Zoologici Fennici, 22, 91–104.

[ece37214-bib-0036] Lambert, T. D. , Adler, G. H. , Riveros, C. M. , Lopez, L. , Ascanio, R. , & Terborgh, J. (2003). Rodents on tropical land‐bridge islands. Journal of Zoology London, 260, 179–187. 10.1017/S0952836903003637

[ece37214-bib-0037] Lambert, T. D. , Malcom, J. R. , & Zimmerman, B. L. (2006). Amazonian small mammal abundances in relation to habitat structure and resource abundance. Journal of Mammalogy, 87(4), 766–776. 10.1644/05-MAMM-A-261R1.1

[ece37214-bib-0038] Leirs, H. , Stenseth, N. C. , Nichols, J. D. , Hines, J. E. , Verhagen, R. , & Verheyen, W. (1997). Stochastic seasonality and nonlinear density‐dependent factors regulate population size in an African rodent. Nature, 389(September), 176–180. 10.1038/38271 9296494

[ece37214-bib-0039] Leirs, H. , Verhagen, R. , & Verheyen, W. (1994). The basis of reproductive seasonality in Mastomys rats (Rodentia: Muridae) in Tanzania. Journal of Tropical Ecology, 10, 55–66.

[ece37214-bib-0040] Leirs, H. , Verheyen, W. , Michiels, M. , Verhagen, R. , & Stuyck, J. (1989). The relation between rainfall and the breeding season of *Mastomys natalensis* (Smith, 1834) in Morogoro, Tanzania. Annales De La Société Royale Zoologique De Belgique, 119, 59–64.

[ece37214-bib-0041] Makundi, R. H. , Massawe, A. W. , & Mulungu, L. S. (2006). Breeding seasonality and population dynamics of three rodent species in the Magamba Forest Reserve, Western Usambara Mountains, north‐east Tanzania. African Journal of Ecology, 45, 17–21. 10.1111/j.1365-2028.2006.00667.x

[ece37214-bib-0042] Mariën, J. , Sluydts, V. , Borremans, B. , Gryseels, S. , Vanden Broecke, B. , Sabuni, C. A. , Katakweba, A. A. S. , Mulungu, L. S. , Günther, S. , de Bellocq, J. G. , Massawe, A. W. , & Leirs, H. (2018). Arenavirus infection correlates with lower survival of its natural rodent host in a long‐term capture‐mark‐recapture study. Parasites & Vectors, 11(1), 90. 10.1186/s13071-018-2674-2 29422075 PMC5806307

[ece37214-bib-0043] Mayamba, A. , Broecke, B. V. , Leirs, H. , Isabirye, B. E. , Byamungu, R. M. , Nakiyemba, A. , Isabirye, M. , Kifumba, D. , Massawe, A. W. , Kimaro, D. N. , Mdangi, M. E. , & Mulungu, L. S. (2019). Fitness of the pestiferous small rodent *Mastomys natalensis* in an agroecosystem in Mayuge district, Lake Victoria Crescent, Uganda. Mammalia, 84(4), 344–353. 10.1515/mammalia-2019-0101

[ece37214-bib-0044] Monadjem, A. , Taylor, P. J. , Denys, C. , & Cotterill, F. P. D. (2015). Rodents of Sub‐Saharan Africa. A biogeographic and taxonomic synthesis. Walter de Gruyter.

[ece37214-bib-0045] Mulungu, L. S. (2017). Chapter 15: Control of rodent pests in maize cultivation: The case of Africa. In D. Watson (Ed.), Achieving sustainable maize cultivation (Vol. 2, pp. 317–337). Francis Dodds: Burleigh Dodds Science Publishing. 10.19103/AS.2016.0002.18

[ece37214-bib-0046] Myers, N. , Mittermeier, R. A. , Mittermeier, C. G. , Fonseca, G. A. B. , & Kent, J. (2000). Biodiversity hotspots for conservation priorities. Nature, 403, 853–858. 10.1038/35002501 10706275

[ece37214-bib-0047] Norrdahl, K. , & Korpimaki, E. (1998). Does mobility or sex of voles affect risk of predation by mammalian predators? Ecology, 79(1), 226–232. 10.1890/0012-9658(1998)079[0226:DMOSOV]2.0.CO;2

[ece37214-bib-0048] Ochoa, J. G. (2000). Efectos de la Extraccion de Maderas sobre la Diversidad de Mamiferos Pequeiios en Bosques de Tierras Bajas de la Guayana Venezolana. Biotropica, 32(1), 146–164.

[ece37214-bib-0049] Oli, M. K. , & Dobson, F. S. (1999). Population cycles in small mammals: The role of age at sexual maturity. Oikos, 86, 557–565. 10.2307/3546660

[ece37214-bib-0050] Ostfeld, R. S. (1985). Limiting resources and territoriality in microtine rodents. The American Naturalist, 126(1), 1–15. 10.1086/284391

[ece37214-bib-0051] Paradis, E. , Guedon, G. , & Pradel, R. (1993). Estimation of sex‐ and age‐related survival rates in a microtine population. Journal of Wildlife Management, 57(1), 1–16. 10.2307/3809012

[ece37214-bib-0052] Pocock, M. J. O. , Frantz, A. C. , Cowan, D. P. , White, P. C. L. , & Searle, J. B. (2004). Tapering bias inherent in Minimum Number Alive (MNA) population indices. Journal of Mammalogy, 85(5), 959–962. 10.1644/BPR-023

[ece37214-bib-0053] Pradel, R. , Wintrebert, C. M. A. , & Gimenez, O. (2003). A proposal for a goodness‐of‐fit test to the Arnason‐Schwarz multisite capture‐recapture model. Biometrics, 59(1), 43–53. 10.1111/1541-0420.00006 12762440

[ece37214-bib-0054] Previtali, M. A. , Lehmer, E. M. , Pearce‐Duvet, J. M. C. , Jones, J. D. , Clay, C. A. , Wood, B. A. , Ely, P. W. , Laverty, S. M. , & Dearing, M. D. (2010). Roles of human disturbance, precipitation, and a pathogen on the survival and reproductive probabilities of deer mice. Ecology, 91(2), 582–592. 10.1890/08-2308.1 20392022

[ece37214-bib-0055] R Core Team (2013). R: A language and environment for statistical computing. R Foundation for Statistical Computing.

[ece37214-bib-0056] Rovero, F. , Menegon, M. , Fjeldså, J. , Collett, L. , Doggart, N. , Leonard, C. , Norton, G. , Owen, N. , Perkin, A. , Spitale, D. , Ahrends, A. , & Burgess, N. D. (2014). Targeted vertebrate surveys enhance the faunal importance and improve explanatory models within the Eastern Arc Mountains of Kenya and Tanzania. Diversity and Distributions, 20, 1438–1449. 10.1111/ddi.12246

[ece37214-bib-0057] Sikes, R. S. ; Animal Care and Use Committee of the American Society of Mammalogists (2016). 2016 Guidelines of the American Society of Mammalogists for the use of wild mammals in research and education. Journal of Mammalogy, 97(3), 663–688. 10.1093/jmammal/gyw078 29692469 PMC5909806

[ece37214-bib-0058] Sluydts, V. , Crespin, L. , Davis, S. , & Lima, M. (2007). Survival and maturation rates of the African rodent, *Mastomys natalensis*: Density‐dependence and rainfall. Integrative Zoology, 2, 220–232. 10.1111/j.1749-4877.2007.00065.x 21396039

[ece37214-bib-0059] Swanepoel, L. H. , Swanepoel, C. M. , Brown, P. R. , Eiseb, S. J. , Goodman, S. M. , Keith, M. , Belmain, S. R. (2017). A systematic review of rodent pest research in Afro‐Malagasy small‐holder farming systems: Are we asking the right questions? PLoS One, 12(3), 1–20. 10.1371/journal.pone.0174554 PMC5373544 28358899

[ece37214-bib-0060] Taylor, K. D. , & Green, M. G. (1976). The influence of rainfall on diet and reproduction in four African rodent species. Journal of Zoology, London, 180, 367–389. 10.1111/j.1469-7998.1976.tb04683.x 1035173

[ece37214-bib-0061] Van Horne, B. (1983). Density as a misleading indicator of habitat quality. The Journal of Wildlife Management, 47(4), 893–901. 10.2307/3808148

[ece37214-bib-0062] Zhang, H. , Wang, Y. , & Zhang, Z. (2009). Domestic goat grazing disturbance enhances tree seed removal and caching by small rodents in a warm‐temperate deciduous forest in China. Wildlife Research, 36, 610–616. 10.1071/WR09001

