# Supplementary figures and images for: Effects of forest disturbance on the fitness of an endemic rodent in a biodiversity hotspot
Source: Ecol Evol. 2021 Feb 3;11(5):2391–401. doi: 10.1002/ece3.7214 (PMC7920783; doi:10.1002/ece3.7214)

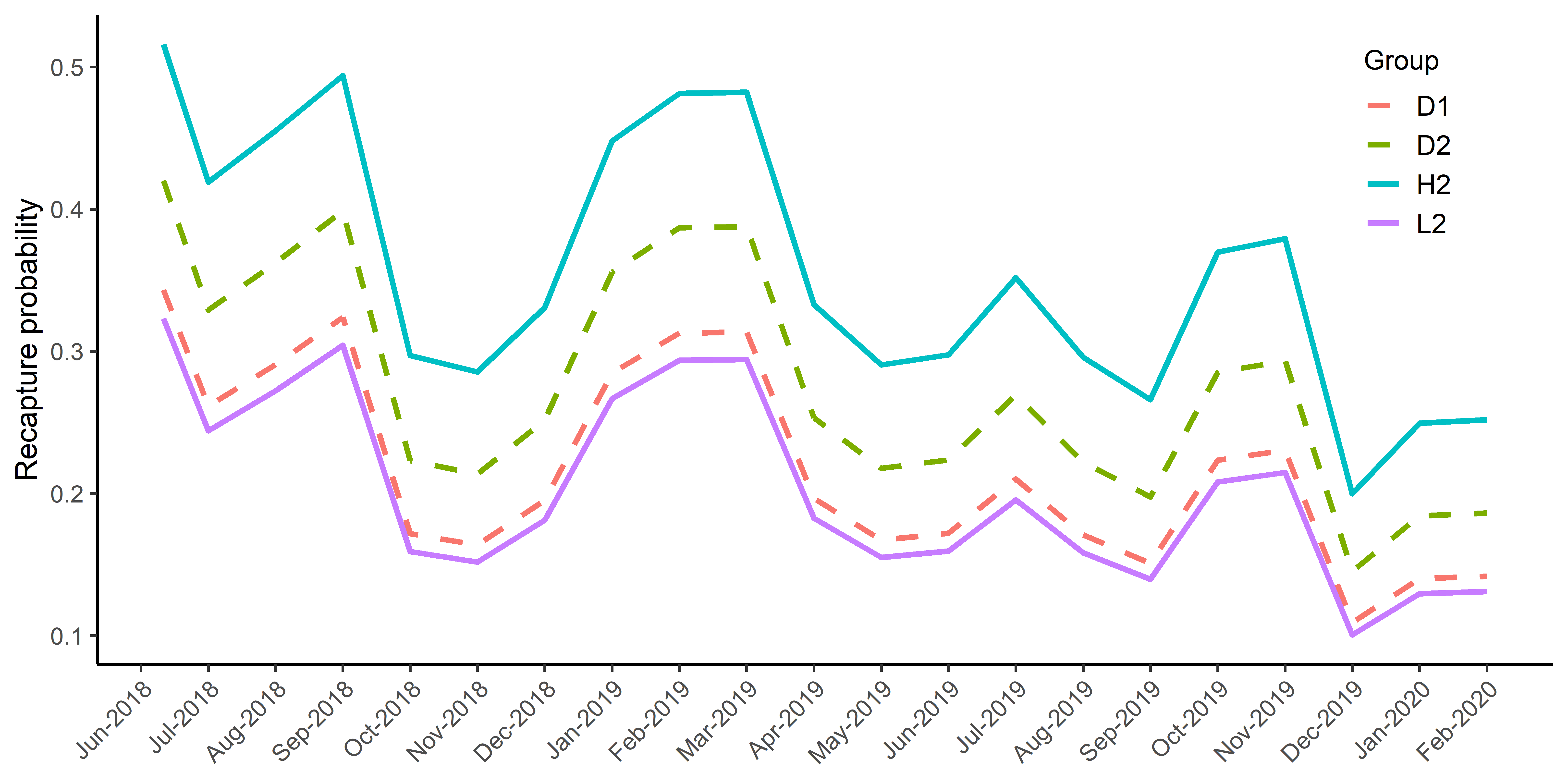

Supplement: Supplementary file 1 — Figure S1 [file ECE3-11-2391-s001.png]

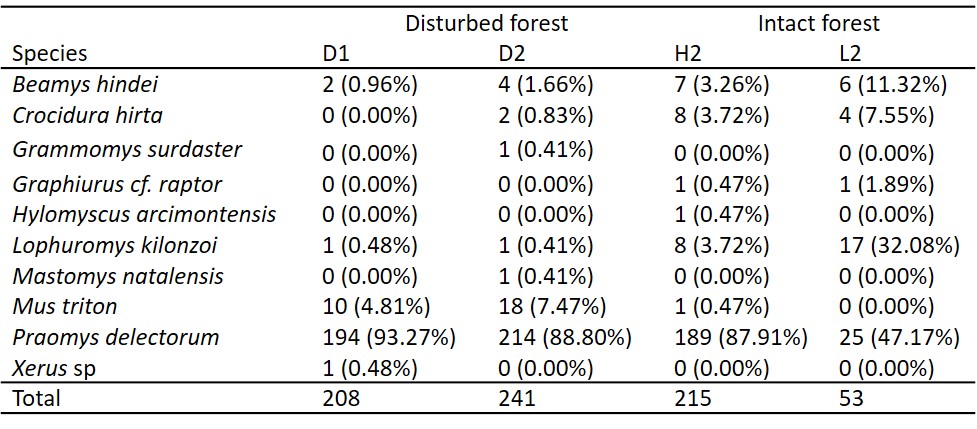

Supplement: Supplementary file 2 — Table S1 [file ECE3-11-2391-s002.jpg]
