# Supplementary material for: Effects of forest disturbance on the fitness of an endemic rodent in a biodiversity hotspot
Source: Ecol Evol. 2021 Feb 3;11(5):2391–401. doi: 10.1002/ece3.7214 (PMC7920783; doi:10.1002/ece3.7214)
Supplement: Supplementary file 3 — Supplementary Material [file ECE3-11-2391-s003.docx]

**Supplementary information**

**Table 1**: Species composition of all captured small mammals in the disturbed (fields D1 & D2) and intact forest (fields H2 & L2). The total number of animals and the percentage for each species is given for each field.

|  | Disturbed forest | | Intact forest | |
| --- | --- | --- | --- | --- |
| Species | D1 | D2 | H2 | L2 |
| *Beamys hindei* | 2 (0.96%) | 4 (1.66%) | 7 (3.26%) | 6 (11.32%) |
| *Crocidura hirta* | 0 (0.00%) | 2 (0.83%) | 8 (3.72%) | 4 (7.55%) |
| *Grammomys surdaster* | 0 (0.00%) | 1 (0.41%) | 0 (0.00%) | 0 (0.00%) |
| *Graphiurus cf. raptor* | 0 (0.00%) | 0 (0.00%) | 1 (0.47%) | 1 (1.89%) |
| *Hylomyscus arcimontensis* | 0 (0.00%) | 0 (0.00%) | 1 (0.47%) | 0 (0.00%) |
| *Lophuromys kilonzoi* | 1 (0.48%) | 1 (0.41%) | 8 (3.72%) | 17 (32.08%) |
| *Mastomys natalensis* | 0 (0.00%) | 1 (0.41%) | 0 (0.00%) | 0 (0.00%) |
| *Mus triton* | 10 (4.81%) | 18 (7.47%) | 1 (0.47%) | 0 (0.00%) |
| *Praomys delectorum* | 194 (93.27%) | 214 (88.80%) | 189 (87.91%) | 25 (47.17%) |
| *Xerus* sp | 1 (0.48%) | 0 (0.00%) | 0 (0.00%) | 0 (0.00%) |
| Total | 208 | 241 | 215 | 53 |


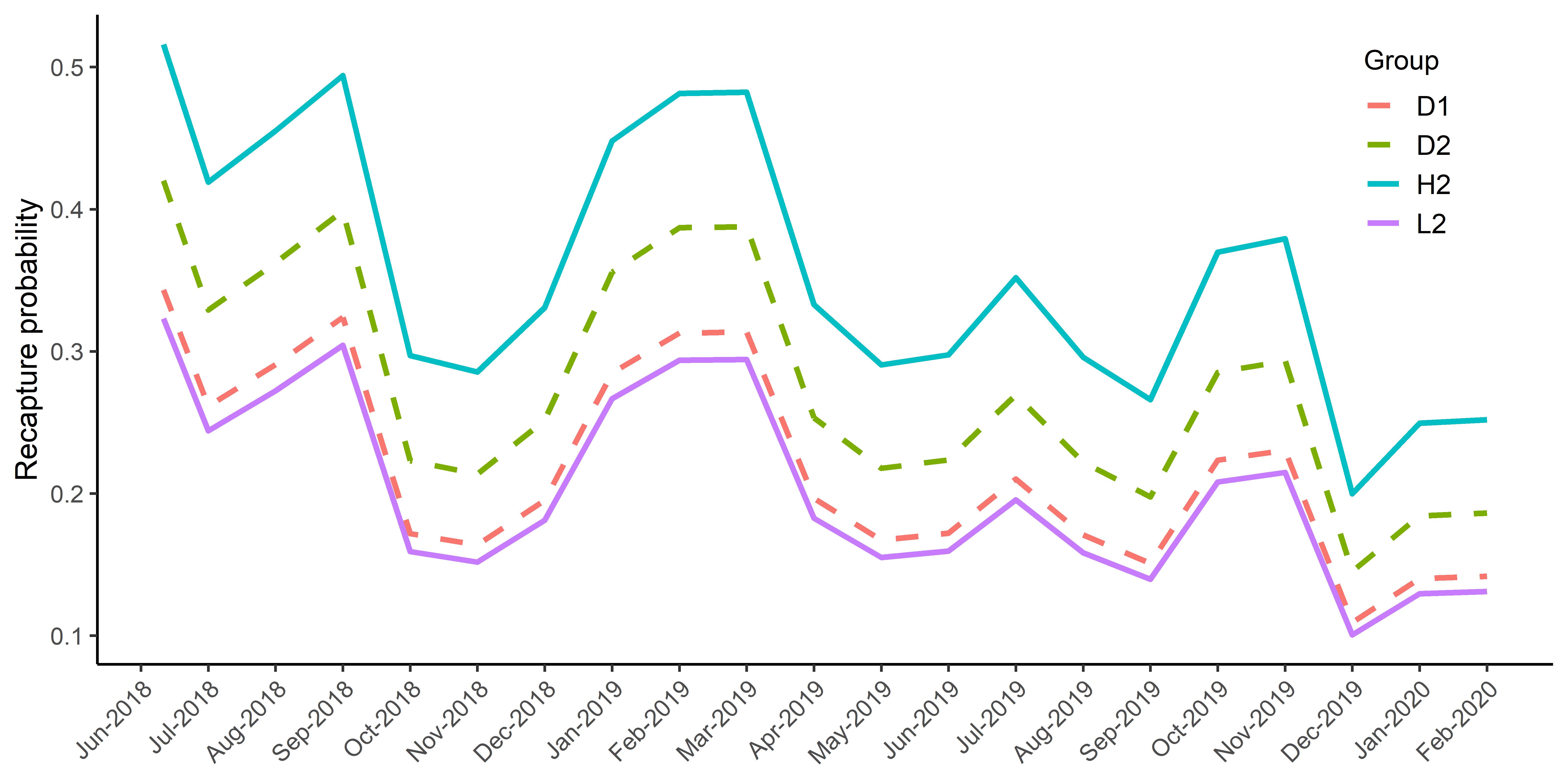


**Figure 1**: Recapture probabilities in both disturbed forests (dashed lines; field D1: red and D2: green) and intact forests (solid lines; field H2: blue and L2: purple) over the different sessions.
